# Associations of life’s essential 8 with extent of multi-territorial atherosclerotic plaques and stenosis: a cross-sectional study

**DOI:** 10.1186/s12877-024-05119-6

**Published:** 2024-06-07

**Authors:** Yanli Zhang, Dandan Liu, Xueli Cai, Aoming Jin, Lerong Mei, Jing Jing, Suying Wang, Xia Meng, Shan Li, Mengxing Wang, Hongyi Yan, Tiemin Wei, Yongjun Wang, Yuesong Pan

**Affiliations:** 1 Department of Neurology, Beijing Tiantan Hospital, Capital Medical University, Beijing, China; 2 China National Clinical Research Center for Neurological Diseases, Beijing, China; 3 Department of Neurology, The Fifth Affiliated Hospital of Wenzhou Medical University, Lishui, China; 4 Cerebrovascular Research Lab, The Fifth Affiliated Hospital of Wenzhou Medical University, Lishui, China; 5 Department of Cardiology, The Fifth Affiliated Hospital of Wenzhou Medical University, Lishui, China; 6 Advanced Innovation Center for Human Brain Protection, Capital Medical University, Beijing, China; 7 Research Unit of Artificial Intelligence in Cerebrovascular Disease, Chinese Academy of Medical Sciences, Beijing, China; 8 Center for Excellence in Brain Science and Intelligence Technology, Chinese Academy of Sciences, Shanghai, China

**Keywords:** Life’s essential 8, Atherosclerosis, Cardiovascular disease, Plaque, Stenosis

## Abstract

**Background:**

Life’s Essential 8 (LE8), the recently updated construct for quantifying cardiovascular health, is related to the risks of cardiovascular events. The present study aimed to evaluate associations of LE8 score with the multi-territorial extent of atherosclerosis in a community-dwelling population.

**Methods:**

Data were derived from the baseline cross-sectional survey of the PolyvasculaR Evaluation for Cognitive Impairment and vaScular Events (PRECISE) study in Lishui City. The LE8 included overall, medical and behavior LE8 scores, and were categorized as low (< 60), moderate (60-<80), and high (≥ 80) groups. Vascular magnetic resonance imaging was used to evaluate intracranial and extracranial arteries; thoracoabdominal computed tomography angiography to evaluate coronary, subclavian, aorta, renal, ilio-femoral arteries; and ankle-brachial index to evaluate peripheral arteries. The presence of atherosclerotic plaque or stenosis in any territory was defined as plaque or vascular stenosis with 1 territory affected or more in these arteries. The extent of atherosclerotic plaques or stenosis was assessed according to the number of these 8 vascular sites affected, and graded as four grades (none, single territory, 2–3 territories, 4–8 territories).

**Results:**

Of 3065 included participants, the average age was 61.2 ± 6.7 years, and 53.5% were women (*n* = 1639). The moderate and high overall LE8 groups were associated with lower extent of multi-territorial plaques [common odds ratio (cOR) 0.44, 95% confidence interval (CI), 0.35–0.55; cOR 0.16, 95%CI, 0.12–0.21; respectively] and stenosis (cOR 0.51, 95%CI, 0.42–0.62; cOR 0.16, 95%CI, 0.12–0.21; respectively) after adjustment for potential covariates. Similar results were observed for medical LE8 score with the extent of multi-territorial plaques and stenosis (*P* < 0.05). We also found the association between behavior LE8 score and the extent of multi-territorial stenosis (*P* < 0.05).

**Conclusions:**

The higher LE8 scores, indicating healthier lifestyle, were associated with lower presence and extent of atherosclerotic plaque and stenosis in southern Chinese adults. Prospective studies are needed to further validate these findings.

**Supplementary Information:**

The online version contains supplementary material available at 10.1186/s12877-024-05119-6.

## Introduction

Atherosclerosis is the leading cause of cardiovascular death and disability worldwide [1]. It was reported that approximately 90% of the asymptomatic Chinese community residents had the presence of atherosclerotic plaques, with nearly 80% in multivessel territories, and 38.5% had arterial stenosis in at least one vascular bed [2, 3]. Previous studies had demonstrated that patients with multi-territorial atherosclerosis had poorer clinical outcomes of major adverse cardiac and cerebrovascular events than those with only 1 vascular territory affected.[4] It is essential to assess the risk of multi-territorial atherosclerosis in the asymptomatic subclinical phase and timely take actions to arrest disease development.[5]

Most previous research assessed cardiovascular disease risk by specific risk factors, which portend a worse prognosis. The American Heart Association (AHA) issued Life’s Essential 8 (LE8) score recently, which emphasizes more ideal (higher) LE8 score have been associated with better cardiovascular health (CVH) [6, 7]. Previous studies have demonstrated that an improving LE8 score is inversely associated with the risks of atherosclerosis cardiovascular disease (ASCVD) [8]. Furthermore, better CVH has been linked to less preclinical atherosclerosis in carotid and coronary arteries [9, 10]. However, most research focused intensively on patients with single or few atherosclerosis-affected territories [11–13]. There are limited data on the relationship between ideal LE8 score and comprehensive assessment of extent of multi-territorial atherosclerosis in the arterial system with intracranial, coronary, and peripheral vascular territories among the asymptomatic community population.

In this cross-sectional study, we evaluated the association of LE8 score with the presence and extent of multi-territorial atherosclerotic plaque and stenosis in a community population based on the baseline survey of the Polyvascular Evaluation for Cognitive Impairment and vaScular Events (PRECISE) study.

## Methods

### Study design and participants

Details of the rationale, design and baseline data for the PRECISE study have been described previously [14]. In brief, the PRECISE study is a prospective community-based study, aimed to investigate the prevalence of polyvascular lesions, the progression rate of plaque, and the association between polyvascular lesions and further events in Chinese older adults. From May 2017 to September 2019, a total of 3067 participants aged 50–75 from Lishui City were recruited in the PRECISE study. Exclusion criteria included contraindications for computed tomography angiography (CTA) and magnetic resonance imaging (MRI), life expectancy of less than 4 years, and mental diseases. All participants were comprehensively evaluated for multi-territorial atherosclerotic plaques and stenosis using thoracoabdominal CTA, vascular MRI, and ankle-brachial index (ABI) at baseline. Written informed consent was given by each participant. The Ethics Committee approved the study protocol of Beijing Tiantan Hospital (IRB Approval No. KY2017-010-01) and Lishui Hospital (IRB Approval No. 2016-42).

### Baseline data collection

Baseline data were obtained through face-to-face interview based on standardized questionnaires, which included demographics, medical history, laboratory features and medical treatment. Hypertension was defined as self-reported previously diagnosed hypertension by physicians or current use of antihypertensive treatment or systolic blood pressure ≥ 140 mmHg or diastolic blood pressure ≥ 90 mmHg [15, 16]. Diabetes mellitus was defined as self-reported previously diagnosed diabetes or current use of antidiabetic treatment or fasting plasma glucose ≥ 7.0 mmol/L or 2-hour post-load glucose ≥ 11.1 mmol/L or hemoglobin A1c ≥ 6.5% [17]. Dyslipidemia was defined as self-reported previously diagnosed dyslipidemia or total cholesterol (TC) ≥ 6.24 mmol/L or low-density lipoprotein cholesterol (LDL-C) ≥ 4.16 mmol/L or high-density lipoprotein cholesterol < 1.04 mmol/L [18]. Use of antihypertensive, antidiabetic, lipid-lowering, antiplatelet and anticoagulant treatments were self-reported.

### Measurement of LE8

The LE8 scoring algorithm consists of 4 medical metrics (body mass index [BMI], blood lipids, blood glucose and blood pressure) and 4 behavioral metrics (diet, physical activity, nicotine exposure, and sleep duration). Details of how the categories were defined are presented in Supplemental Table 1. Briefly, each of the 8 metrics was scored ranging from 0 to 100 points, and the higher points indicated a healthier lifestyle [6]. In this study, medical and behavior LE8 scores were used to further investigate the association of LE8 subscales with the extent of multi-territorial atherosclerotic plaques and stenosis. The overall, medical and behavior LE8 scores were calculated by the unweighted average of components included, and then classified as low (< 60), moderate (60-<80) and high (≥ 80) groups.

Data on vegetables, fruit, meat, poultry, aquatic and alcohol were collected to evaluate the diet metric according to the Mediterranean Eating Pattern for Americans (MEPA). Frequency and duration of activity were collected to assess the grade of physical activity. Sleep duration was calculated according to sleep time and wake-up time. Besides, BMI was defined as weight (kg) divided by the square of height (m) [19]. Blood pressure was measured three times and determined as the average of the second and third consecutive measurements. Fasting (12-hour) blood samples were collected and sent to central laboratory for the determination of blood lipids, plasma glucose and hemoglobin A1c.

### Measurements of vascular MRI

At baseline, intracranial artery and extracranial carotid artery were conducted for all the participants on a 3T scanner (Ingenia 3.0T; Philips, Best, the Netherlands). Patients were scanned in a supine, head-first position. The imaging sequences included 3-dimensional (3D) time-of-flight, 3-D isotropic high-resolution black-blood T1w vessel wall imaging, and simultaneous non-contrast angiography and intraplaque hemorrhage imaging. The evaluated intracranial arterial segments included the distal internal carotid artery, A_1_ and A_2_ segments of the anterior cerebral artery, M_1_ and M_2_ segments of the middle cerebral artery, P_1_ and P_2_ segments of the posterior cerebral artery, basilar artery, and V_4_ segments of the vertebral artery. The extracranial arterial segments included the proximal internal carotid artery, V_1_, V_2_, and V_3_ segments of the vertebral artery, and the common carotid artery. The presence of intra-/extra-cranial atherosclerotic plaque was defined as eccentric wall thickening with or without luminal stenosis identified on the 3D T1w VWI images in comparison to the corresponding location on the 3D TOF MR angiogram [20], and the presence of intra-/extra-cranial atherosclerotic stenosis was defined as stenosis 50–99% or occlusion in the intra-/extra-cranial arteries [21, 22]. Imaging assessment was performed by 2 well-trained raters (D.Y and H.L) who were blinded to participants’ clinical data. Inconsistent results were finally assessed by another senior neurologist (J.J) who was blinded to initial results. The kappa coefficients of MRI markers between raters were 0.97 for the presence of intracranial plaque, 0.79 for intracranial artery stenosis ≥ 50%, 0.94 for extracranial plaque, and 0.86 for extracranial artery stenosis ≥ 50%.

### Measurements of thoracoabdominal CTA

Atherosclerotic plaques and stenosis in coronary, subclavian, aorta, renal, and iliofemoral arteries were conducted for all the participants on a dual-source CT scanner (SOMATOM Force, Siemens Healthineers, Forchheim, Germany) at baseline survey. All participants were injected with the contrast medium iodixanol (320 mg I/mL [Visipaque, GE Healthcare]) for contrast-enhanced thoracoabdominal CTA based on a standard operating procedure. We used a multitask deep learning network to reconstruct the 3-D anatomical geometry of the input CTA images, and calculated quantitative results for plaque and stenosis. Atherosclerosis was classified by professionals based on the 3-D geometry, quantitative results, and CTA image. The evaluated coronary arterial segments included left main, left descending, left circumflex, obtuse margin, diagonal, septal branch, right coronary, posterior branches, and right posterior descending arteries. The subclavian arterial segments included left and right arteries. The aorta arterial segments included arcus aortae and abdominal aorta arteries. The renal arterial segments included left and right arteries. The iliofemoral arterial segments included common iliac, internal iliac and external iliofemoral arteries. Atherosclerotic plaque on CTA was assessed by tissue structures > 1 mm [2] within or adjacent to the coronary artery lumen [21], and atherosclerotic stenosis was defined as stenosis 50–99% or occlusion in thoracoabdominal arteries [22]. Imaging assessment was performed by 2 well-trained raters (Z.Z.Q. and Z.Z.X.) who were blinded to participants’ clinical information. The kappa coefficients of CTA markers between raters were 0.96 for the presence of plaque, and 0.72 for artery stenosis ≥ 50%.

### Measurements of ABI

We measured ABI to assess atherosclerosis in peripheral arteries using a Doppler ultrasound device (Huntleigh Health Care Ltd) at baseline survey. ABI was calculated as the ratio of ankle SBP divided by arm SBP. An ABI ≤ 0.90 was considered abnormal in peripheral arteries by the American College of Cardiology Foundation/AHA guideline [23]. 

### Extent of multi-territorial atherosclerotic plaques and stenosis

The multi-territorial extent of atherosclerotic plaques and stenosis was defined according to the number of 8 vascular sites affected (intracranial, extracranial, coronary, subclavian, aorta, renal, iliofemoral or peripheral arteries), and further graded as none, single territory, 2–3 territories, 4–8 territories affected [3]. We also defined the presence of atherosclerotic plaque or stenosis in any territory as plaque or vascular stenosis with 1 territory affected or more in these arteries.

### Statistical analysis

We described categorical variables by frequency with percentage and continuous variables by mean with SD or median with interquartile. We used the chi-square test or Fisher exact test for categorical data of the participants and ANOVA or Kruskal-Wallis test for continuous data. We first evaluated the associations of LE8 score with the presence of atherosclerotic plaques and stenosis in any territory using binary logistic regression models and calculated odds ratios (ORs) and the corresponding 95% confidence intervals (CIs). In addition, we also assessed the associations of LE8 score with the extent of atherosclerotic plaques and stenosis using ordinary logistic regression models and calculated common odds ratios (cORs) and the corresponding 95% CIs. The multivariable models were adjusted for age, gender, current drinking, estimated glomerular filtration rate, antiplatelet and anticoagulant drugs. To assess the robustness of the findings, sensitivity analyses were performed in participants without history of ASCVD, which was assessed by previous stroke or myocardial infarction. In addition, the abilities to discriminate the presence of atherosclerotic plaque and stenosis in any territory were evaluated in receiver operator curves analysis and quantified by the area under the curve. Statistical analyses were conducted using SAS software, version 9.4 (SAS Institute, Inc, Cary, NC). All statistical tests were 2-sided, and *P* < 0.05 was considered statistically significant.

## Results

### Baseline characteristics

A total of 3067 community-dwelling adults participated in the PRECISE study. After the exclusion of participants with missing data of blood lipids (*n* = 2), 3065 participants were eligible and included in this analysis. Baseline characteristics of the study population stratified by overall LE8 score are presented in Table 1. Among the enrolled participants, the mean age was 61.2 ± 6.7 years, and 53.5% were women (*n* = 1639). Participants with lower LE8 score had more frequent hypertension, diabetes mellitus and dyslipidemia, and higher levels of BMI, TC, LDL-C and fasting plasma glucose (*P* < 0.05).

**Table 1:** Baseline characteristics of participants by overall LE8 score

Variable	Overall(*n* = 3065)	Overall LE8 score			*P* value
		Low (< 60) (*n* = 469)	Moderate (60-<80) (*n* = 2031)	High (≥ 80) (*n* = 565)	
Demographic data					
Age, mean ± SD	61.2 ± 6.7	62.1 ± 6.7	61.5 ± 6.7	59.6 ± 6.4	< 0.001
Female, n (%)	1639 (53.5)	147 (31.3)	1111 (54.7)	381 (67.4)	< 0.001
Current smoking, n (%)	629 (20.5)	237 (50.5)	380 (18.7)	12 (2.1)	< 0.001
Current drinker, n (%)	574 (18.7)	118 (25.2)	387 (19.1)	69 (12.2)	< 0.001
BMI, kg/m^2^, mean ± SD	23.8 ± 3.1	25.4 ± 3.4	23.8 ± 3.0	22.3 ± 2.3	< 0.001
SBP, mmHg, mean ± SD	129.3 ± 16.3	142.1 ± 15.7	129.8 ± 15.0	116.7 ± 12.0	< 0.001
DBP, mmHg, mean ± SD	75.2 ± 9.0	81.7 ± 9.2	75.3 ± 8.4	69.4 ± 7.4	< 0.001
Health diet, MEPA= (5–6), n (%)	319 (10.4)	15 (3.2)	178 (8.8)	126 (22.3)	< 0.001
Health physical activity, > 90 min/per week, n (%)	2974 (97.0)	421 (89.8)	1989 (97.9)	564 (99.8)	< 0.001
Health sleep duration, 7-<9 h/per night, n (%)	1727 (56.4)	164 (35.0)	1158 (57.0)	405 (71.7)	< 0.001
Medical history, n (%)					
Hypertension	1320 (43.1)	352 (75.1)	907 (44.7)	61 (10.8)	< 0.001
Diabetes mellitus	662 (21.6)	203 (43.3)	430 (21.2)	29 (5.1)	< 0.001
Family history of ASCVD	616 (20.1)	98 (20.9)	402 (19.8)	116 (10.8)	0.83
Dyslipidemia	1280 (41.8)	271 (57.8)	875 (43.1)	134 (23.7)	< 0.001
Laboratory feature, mean ± SD					
TC, mmol/L	5.28 ± 0.99	5.73 ± 1.10	5.31 ± 0.96	4.80 ± 0.80	< 0.001
HDL, mmol/L	1.37 ± 0.34	1.28 ± 0.33	1.36 ± 0.33	1.44 ± 0.34	< 0.001
LDL-C, mmol/L	2.78 ± 0.79	3.03 ± 0.89	2.80 ± 0.78	2.46 ± 0.63	< 0.001
FPG, mmol/L	5.96 ± 1.58	6.97 ± 2.79	5.90 ± 1.25	5.33 ± 0.50	< 0.001
HbA1c, mmol/L, mean ± SD	5.94 ± 0.93	6.50 ± 1.51	5.91 ± 0.80	5.56 ± 0.40	< 0.001
eGFR, ml/min per 1.73 m^2^	101.9 ± 12.7	100.1 ± 14.0	101.8 ± 12.8	103.8 ± 11.2	< 0.001
Medication treatment, n (%)					
Antihypertensive	822 (26.8)	213 (45.4)	569 (28.0)	40 (7.1)	< 0.001
Antidiabetic	273 (8.9)	90 (19.2)	176 (8.7)	7 (1.2)	< 0.001
Lipid-lowering	120 (3.9)	27 (5.8)	80 (3.9)	13 (2.3)	0.02
Antiplatelet	80 (2.6)	19 (4.1)	53 (2.6)	8 (1.4)	0.03
Anticoagulant	4 (0.1)	1 (0.2)	3 (0.15)	0 (0.0)	0.60

ASCVD indicates atherosclerotic cardiovascular disease; BMI, body mass index; DBP, diastolic blood pressure; eGFR, estimated glomerular filtration rate; FPG, fasting plasma glucose; HDL-C, high density lipoprotein cholesterol; HbA1c, hemoglobin A1c; LE8, Life’s Essential 8; LDL-C, low density lipoprotein cholesterol; MEPA, Mediterranean Eating Pattern for Americans; SBP, systolic blood pressure; TC, total cholesterol

### Status of LE8 score with multi-territorial plaques and stenosis

**Fig. 1:**
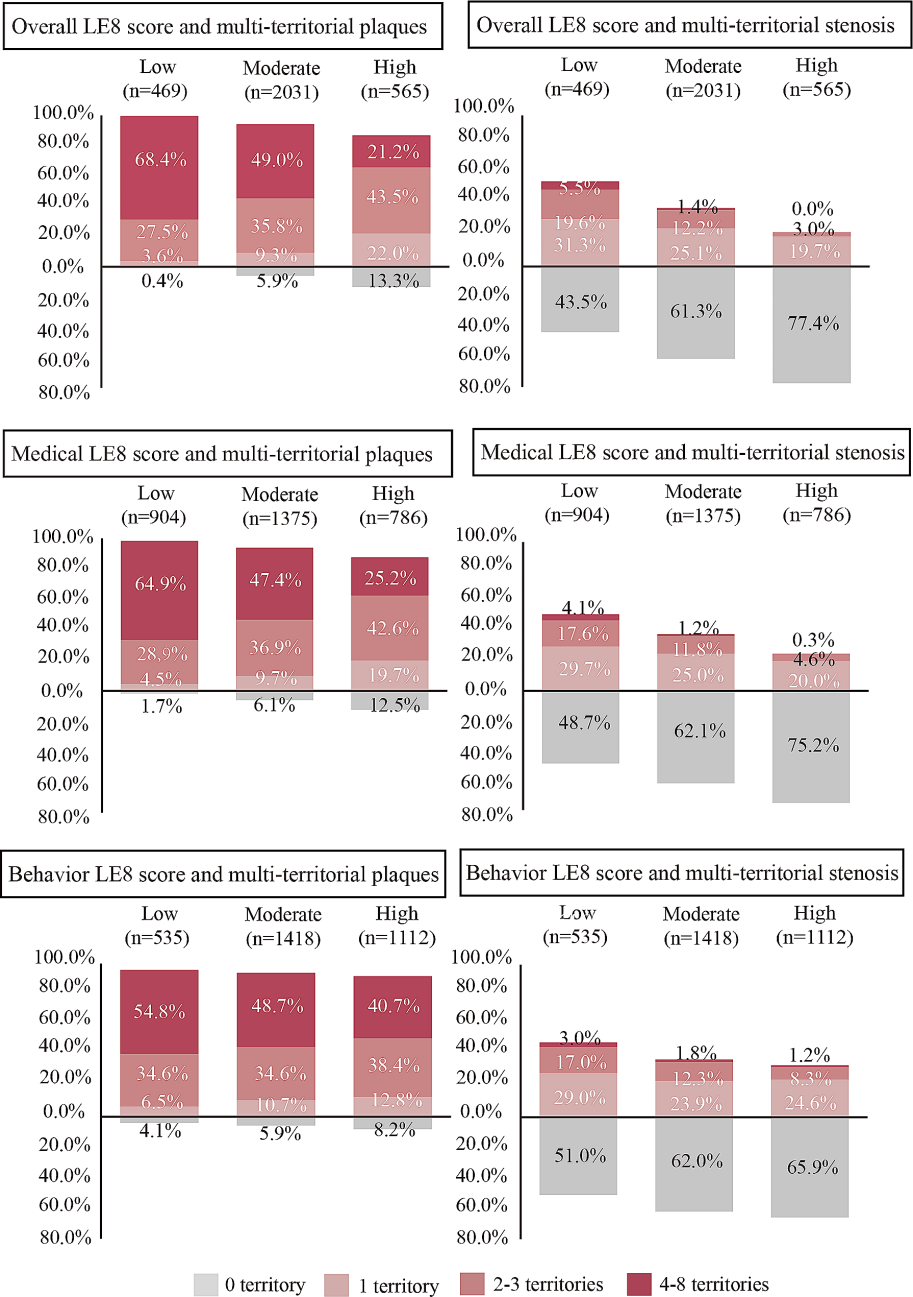
Distribution of the extent of multi-territorial plaque and stenosis according to LE8 categories. LE8 indicates Life’s Essential 8. ^a^ Multi-territorial extent of atherosclerotic plaques and stenosis were graded: none, single territory, 2–3 territories, 4–8 territories; ^b^ Overall, medical and behavior LE8 scores were classified as low (< 60), moderate (60-<80) or high (≥ 80) group

Distributions of the extent of multi-territorial atherosclerotic plaque and stenosis according to overall, medical and behavior LE8 categories were presented in Fig. 1. There were 469 (15.3%) participants in the low group stratified by overall LE8 score, 2031 (66.3%) in the moderate group and 565 (18.4%) in the high group. The proportions were 99.6% with atherosclerotic plaque in any artery among those at low group of overall LE8 score, including 3.6% with 1 territory, 27.5% with 2–3 territories, and 68.4% with 4–8 territories. Whereas, still 86.7% of individuals have any atherosclerotic plaque among those at high group of overall LE8 score, with 64.7% having multi-territorial atherosclerosis. In individual vascular territory, we found that atherosclerotic plaque and stenosis were mostly detected in ilio-femoral artery (Figures S1 and S2).

**Fig. 2:**
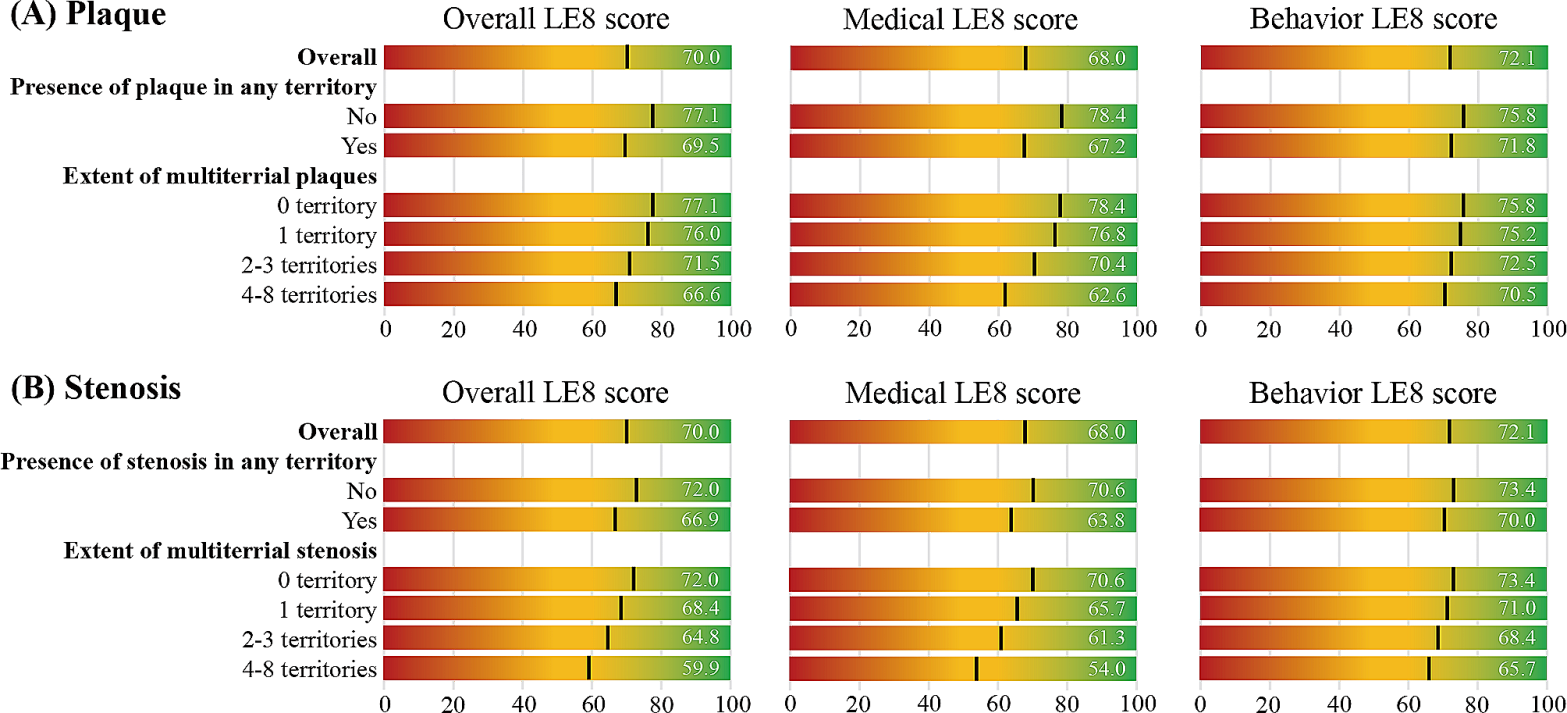
Overall, behavior and medical LE8 scores with multi-territorial plaques and stenosis. LE8 indicated Life’s Essential 8. ^a^ Values are mean scores for overall, presence of plaque or stenosis in any territory, and extent of multi-territorial plaques or stenosis

Figure 2 and S3 display the mean LE8 score (possible range, 0-100) for participants with overall and by the presence of plaque or stenosis in any territory, the extent of multi-territorial plaques or stenosis, and the presence of plaque or stenosis in individual territory. We observed that the overall mean LE8 score was 70, which is moderate CVH. In addition, overall, medical and behavior LE8 scores were lower in participants with the presence of atherosclerotic plaques or stenosis than those without being affected. Compared with the lower extent of atherosclerotic lesions, we also observed lower LE8 scores in participants with a higher extent of polyvascular atherosclerotic plaque or stenosis.

### Association of LE8 score and multi-territorial plaques and stenosis

**Fig. 3:**
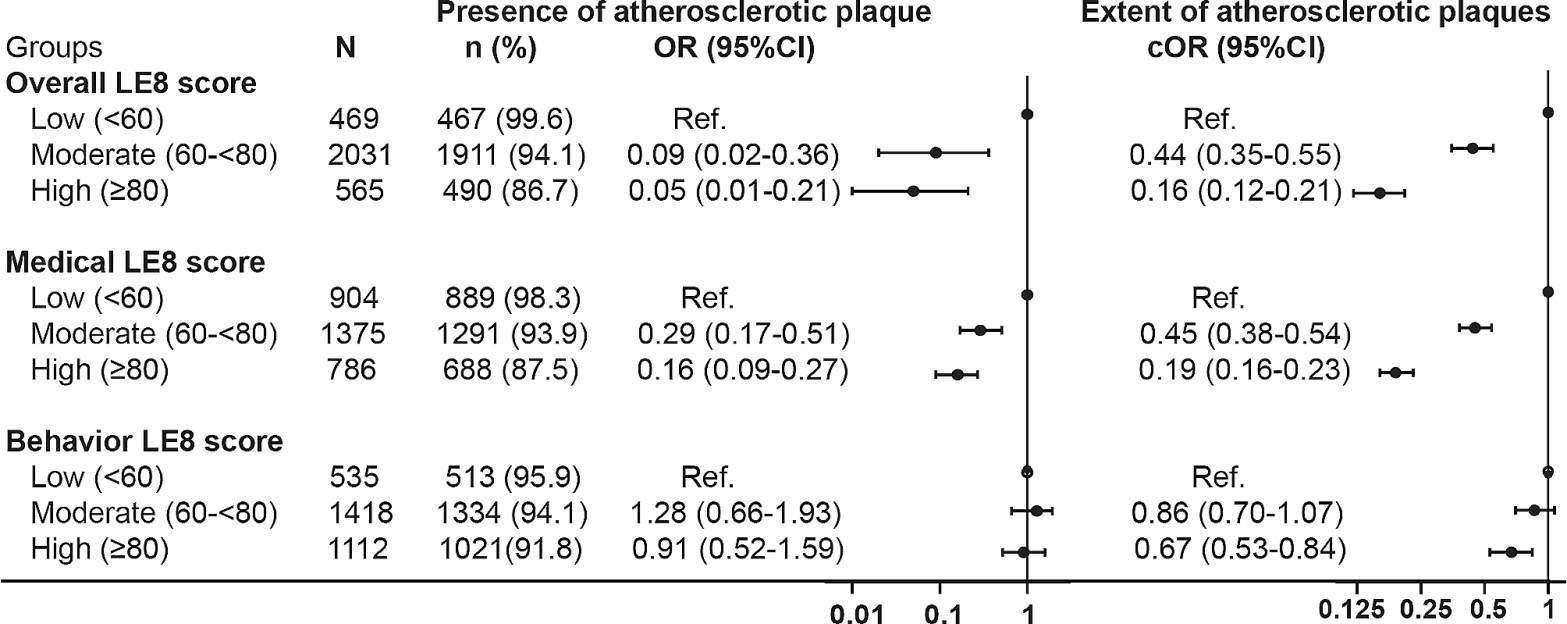
Associations of LE8 with the extent of atherosclerotic plaques. OR indicates odds ratio; cOR, common odds ratio; CI, confidence interval; LE8, Life’s Essential 8. ^a^ Multi-territorial extent of atherosclerotic plaques was graded: none, single territory, 2–3 territories, 4–8 territories; ^b^ The multivariable model was adjusted for age, gender, current drinking, estimated glomerular filtration rate, antiplatelet and anticoagulants drugs

**Fig. 4:**
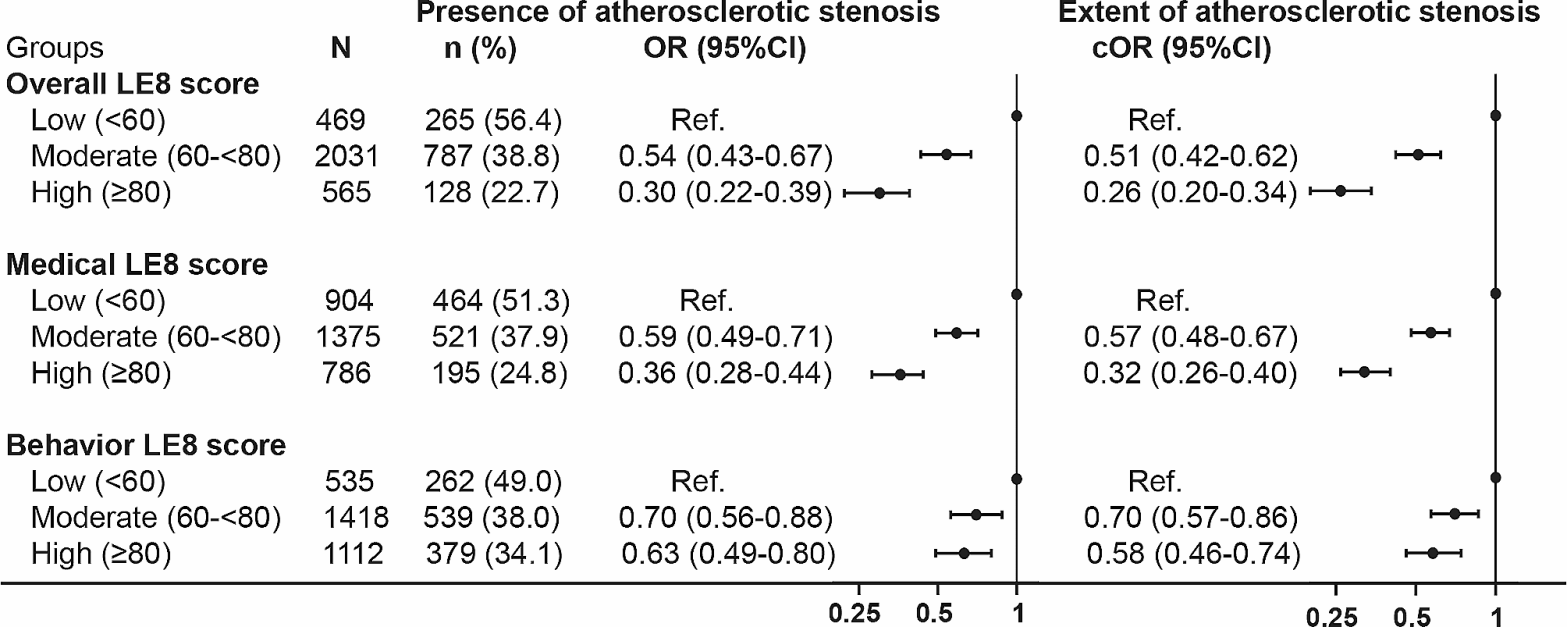
Associations of LE8 with the extent of atherosclerotic stenosis. OR indicates odds ratio; cOR, common odds ratio; CI, confidence interval; LE8, Life’s Essential 8. ^a^ Multi-territorial extent of atherosclerotic plaques was graded: none, single territory, 2–3 territories, 4–8 territories; ^b^ The multivariable model was adjusted for age, gender, current drinking, estimated glomerular filtration rate, antiplatelet and anticoagulants drugs

After adjusting for potential covariates, the moderate and high groups of overall LE8 score were associated with lower odds of the presence of plaque in any territory (OR 0.09, 95%CI, 0.02–0.36; OR 0.05, 95%CI, 0.01–0.21; respectively) and the lower extent of multi-territorial plaques (cOR 0.44, 95%CI, 0.35–0.55; cOR 0.16, 95%CI, 0.12–0.21; respectively, Fig. 3) compared with low LE8 groups. We also observed that the associations between moderate and high groups of overall LE8 score and the extent of multi-territorial stenosis (cOR 0.51, 95%CI, 0.42–0.62; cOR 0.26, 95%CI, 0.20–0.34; respectively, Fig. 4). Similar results were observed for the medical and behavior LE8 score with the extent of multi-territorial plaques and stenosis (Figs. 3 and 4). However, associations between behavior LE8 score with the presence of multi-territorial plaque were not found. Furthermore, we also found consistent results when evaluating the relationship between overall and medical LE8 scores and atherosclerotic plaques and stenosis in each vascular territory (Tables S2 and S3). In the sensitivity analysis, we also found overall, medical and behavior LE8 scores were associated with the presence and extent of atherosclerotic stenosis in participants without history of ASCVD (Tables S4). In addition, the area under the curve of the overall, medical and behavior LE8 scores were 0.651, 0.678 and 0.565 for discriminating the presence of atherosclerotic plaque in any territory, and 0.600, 0.612 and 0.553 for discriminating the presence of atherosclerotic stenosis in any territory (Tables S5).

## Discussion

In this large-scale population-based survey of the PRECISE study, we observed that higher overall and medical LE8 scores were associated with lower odds of presence and lower extent of atherosclerotic plaque and stenosis. In addition, participants with lower overall, medical and behavior LE8 scores tend to have more number territories of atherosclerotic plaque or stenosis. Our findings suggested that having a healthier lifestyle may be associated with a decreasing burden of subclinical atherosclerosis among community-dwelling older Chinese adults.

To our knowledge, most previous studies reported that ideal CVH metrics were associated with lower prevalence and risk of cardiovascular diseases and atherosclerotic lesions, which indicated the significance of improvement in CVH status [8, 11, 24–28]. Prospective data from the UK Biobank study and Northern Manhattan Study (NOMAS) showed a steep gradient relationship between ideal CVH with individual cardiovascular diseases [11, 25]. Other studies also reported that ideal CVH plays a virtual role in the reduction of the subclinical atherosclerosis risk [29]. The National Heart, Lung, and Blood Institute Family Heart Study indicated that the number of ideal CVH metrics was inversely related to the calcified atherosclerotic plaque in the coronary arteries [27]. In parallel, previous studies also reported the negative correlation between Life’s Simple 7 metrics and subclinical atherosclerosis in the carotid arteries, which further supports the application of CVH metrics in subclinical prevention [10, 28]. Furthermore, a cohort study in China proved that CVH score was an independent predictor of atherosclerosis progression in the middle-aged and older population [30]. These evidence supported our findings that ideal LE8 score was inversely related to the risk of atherosclerotic lesions. A large population-based study from the Swedish Cardiopulmonary Bioimage Study (SCAPIS) demonstrated that the newly developed LE8 score was inversely associated with carotid plaques and subclinical coronary atherosclerosis [31, 32]. The Mediators of Atherosclerosis in South Asians Living in America (MASALA) Study reported that higher CVH assessed by LE8 score correlated with lower odds of any coronary artery calcium in South Asian American adults [33]. However, since atherosclerosis is a diffuse and systemic disease, evaluation on multi-territorial vascular beds with the advanced high-resolution imaging techniques in our study could comprehensively and accurately reflect the extent of subclinical atherosclerosis in the arterial system, and may find potential cerebrovascular events that cannot be detected when we considered only single territory [3]. As with previous studies, our observational study found similar associations of ideal LE8 score and its subscales with the extent of multi-territorial atherosclerotic plaque and stenosis in the asymptomatic community population. This expands upon prior studies which had shown the relationship of a healthy lifestyle with decreased risk of subclinical atherosclerosis in single or few territories [8]. Previous studies have demonstrated that atherosclerosis is the major cause of cardiovascular disease and stroke [1, 34]. Screening of individuals with atherosclerosis is helpful for the prevention of cardiovascular and cerebrovascular diseases. Therefore, our study indicated that keeping an optimal CVH status or improving the CVH status is essential to alleviate the burden of atherosclerosis and ASCVD. However, we did not observe the association of behavior LE8 score with the presence of atherosclerotic plaque. The most probable reason is that diet and sleep health indirectly affect the formation of atherosclerosis plaque and stenosis and thus have a weaker correlation with subclinical arteriosclerosis [31, 35]. 

The natural history of atherosclerosis includes a protracted asymptomatic phase, which may delay diagnosis and treatment because of their atypical symptoms [36]. Previous studies reported that subclinical carotid atherosclerosis and coronary artery calcification increased the risk of cardiovascular disease events [37, 38]. Renal atherosclerotic stenosis may be associated with resistant hypertension and ischemic nephropathy [39]. What’s more, intracranial atherosclerosis is associated with a high risk of recurrent stroke in the Asian population [40, 41]. These vascular territories were easily overlooked in the routine examination but were of significance for future cardiovascular and cerebrovascular events prevention. Since CVH factors, including diabetes, hypertension, dyslipidemia, diet, physical activity, smoking and sleep duration, are the major modifiable risk factors associated with atherosclerotic disease [40], CVH evaluation of atherosclerosis in its subclinical stage may help increase the ability to predict subsequent risk of complication and promote the primary prevention of ASCVD [3, 8, 36]. Our observational study evaluated overall lifestyle risk of multi-territorial extent of subclinical atherosclerosis using the LE8 score, a comprehensive indicator to quantify CVH, which may provide an index to assess the state of subclinical atherosclerosis health. In this study, participants with lower LE8 scores tend to have more number territories of vascular lesions among community-dwelling older adults, which indicated that promoting healthy lifestyles and keeping an optimal CVH status is vital to alleviate the disease burden of ASCVD. These evidence suggested that more aggressive efforts need to be adopted to help more populations shift toward ideal CVH [42]. Further studies should continue to assess whether improving the LE8 modifiable risk factors can reduce the risk of multi-territorial atherosclerosis.

### Study strengths and limitations

The major strengths of this study are that we comprehensively collected the baseline data, and accurately assessed multi-territorial atherosclerosis using advanced vascular imaging techniques in a largescale community-based population. This study also has several limitations. First, self-reported nicotine exposure and physical activity may result in recall and social desirability biases. Information on diet was collected including green leafy vegetables, fruit, meat, fish, poultry and alcohol according to MEPA, but other dietary components, such as whole grains, nuts, beans, sweets and pasties, and cheese, were missing. Our results may underestimate the diet score of participants and therefore should be interpreted with caution. In addition, some risk factors were self-reported, such as disease history, smoking and drinking status, and medication treatment, which may result in unavoidable memory bias. Second, as a cross-sectional analysis of the baseline survey of the PRECISE cohort, the study design did not allow conclusions on a causal relationship between the ideal LE8 score and the multi-territorial extent of subclinical atherosclerosis. Longitudinal study is needed to validate the causal relationship. Third, different technologies were used to evaluate the atherosclerotic lesions in different arteries, resulting in differences in the sensitivity of detecting atherosclerosis and may affect the comparisons among different vascular territories. Finally, despite being representative in general, potential selection bias remains because most of the participants in the PRECISE study were from one rural area. The findings in this study need to be verified in a larger sample size of the population in the future.

## Conclusions

In conclusion, the higher LE8 score, indicating a healthier lifestyle, was associated with lower odds of the presence and lower extent of atherosclerotic plaque and stenosis in southeastern Chinese adults. In addition, participants with lower LE8 scores tend to have more number territories of vascular lesions. This indicates that maintaining an optimal lifestyle may be of great value in preventing subclinical atherosclerosis. Prospective studies are needed to further validate these results in a large more ethnically diverse cohort.

### Electronic supplementary material

Below is the link to the electronic supplementary material.

Supplementary Material 1

## Data Availability

Data are available to researchers on request for purposes of reproducing the results by directly contacting the corresponding author.

## Abbreviations

LE8: Life’s Essential 8
CVH: Cardiovascular health
ASCVD: Atherosclerosis cardiovascular disease
PRECISE: Polyvascular Evaluation for Cognitive Impairment and vaScular Events
CTA: Computed tomography angiography
MRI: Magnetic resonance imaging
ABI: Ankle-brachial index
TC: Total cholesterol
LDL-C: Low-density lipoprotein cholesterol
BMI: Body mass index
MEPA: The Mediterranean Eating Pattern for Americans
ORs: Odds ratios
cORs: Common odds ratios
CIs: Confidence intervals
