# Supplementary material for: Associations of life’s essential 8 with extent of multi-territorial atherosclerotic plaques and stenosis: a cross-sectional study
Source: BMC Geriatr. 2024 Jun 7;24:503. doi: 10.1186/s12877-024-05119-6 (PMC11157784; doi:10.1186/s12877-024-05119-6)

**Supplemental Material**

**CONTENTS**

**Supplemental Tables**

**Table S1** Definition and scoring approach for the American Heart Association’s Life’s Essential 8 score.

**Table S2** Associations of LE8 with the presence of atherosclerotic plaque in individual vascular territory.

**Table S3** Associations of LE8 with the presence of atherosclerotic stenosis in individual vascular territory.

**Table S4** Subgroup analysis of LE8 with the presence and extent of atherosclerotic stenosis in participants without history of ASCVD.

**Table S5** Receiver operator curve of LE8 for the presence of atherosclerotic plaque and stenosis.

**Supplemental Figure Legends**

**Fig. S1** Distribution of LE8 categories with the presence of atherosclerotic plaque in individual vascular territory.

**Fig. S2** Distribution of LE8 categories with the presence of atherosclerotic stenosis in individual vascular territory.

**Fig. S3** Values of mean scores for LE8 scores with presence of plaque and stenosis.

**SUPPLEMENTAL TABLES**

**Table S1 Definition and scoring approach for the American Heart Association’s Life’s Essential 8 score.**

| **Domain** | **CVH metric** | **Measurement** | **Quantification and Scoring of CVH Metric** | |
| --- | --- | --- | --- | --- |
| **Health factors** | BMI | Body weight (kg) divided by height squared (m^2^) | Points:  100  70  30  15  0 | BMI (kg/m^2^)  <25  25.0–29.9  30.0–34.9  35.0–39.9  ≥40.0 |
|  | Blood lipids | Plasma total and HDL cholesterol with calculation of non–HDL cholesterol | Points:  100  60  40  20  0 | Non–HDL cholesterol (mg/dL)  <130  130–159  160–189  190–219  ≥220 |
|  |  |  | If drug-treated level, subtract 20 points (unless score is 0) | |
|  | Blood glucose | Fasting blood glucose (mg/dL) or Hemoglobin A1c (%) | Points:  100  60  40  30  20  10  0 | Points level:  No history of diabetes and FBG < 100 (or HbA1c< 5.7)  No diabetes and FBG 100-125 (or HbA1c 5.7-6.4) (Pre-diabetes)  Diabetes with HbA1c <7.0  Diabetes with HbA1c 7.0–7.9  Diabetes with HbA1c 8.0–8.9  Diabetes with Hb A1c 9.0–9.9  Diabetes with HbA1c ≥10.0 |
|  | Blood pressure | Systolic and diastolic blood pressure (mm Hg) | Points:  100  75  50  25  0 | Systolic and diastolic BPs (mmHg)  <120/<80 (optimal)  120–129/<80 (elevated)  130–139 or 80–89 (stage 1 hypertension)  140–159 or 90–99  ≥160 or ≥100 |
|  |  |  | If drug-treated level, subtract 20 points (unless score is 0) | |
| **Health Behaviors** | Diet^a^ | Mediterranean Eating Pattern for Americans (MEPA) | Points:  100  80  50  25  0 | MEPA score (points):  6  5  4  3  0-2 |
|  | Physical activity | Self-reported minutes of moderate or vigorous PA per week | Points:  100  90  80  60  40  20  0 | Points Minutes:  ≥150  120 – 149  90-119  60-89  30-59  1-29  0 |
|  | Nicotine exposure | Combustible tobacco used and/or inhaled NDS use; or secondhand smoke exposure | Points:  100  75  50  25  0 | Points Status:  Never smoker  Former smoker, quit ≥5 years  Former smoker, quit 1 -<5 years  Former smoker, quit <1 year, or currently using inhaled NDS  current smoker |
|  |  |  | Subtract 20 points (unless score is 0) for living with active indoor smoker in home | |
|  | Sleep health | Average hours of sleep per night | Points:  100  90  70  40  20  0 | Points level (hours):  7-<9  9-<10  6-<7  5-<6 or ≥10  4-<5  <4 |

CVH indicated cardiovascular disease health; BMI, body mass index; PA, physical activity.

^a^ Diet was calculated by modified MEPA based on green leafy vegetables, fruit, meat, fish, poultry and alcohol.

**Reference**

1. Lloyd-Jones DM, Allen NB, Anderson CAM, et al. Life's Essential 8: Updating and Enhancing the American Heart Association's Construct of Cardiovascular Health: A Presidential Advisory From the American Heart Association. Circulation. 2022;146(5):e18-e43.

**Table S2 Associations of LE8 with the presence of atherosclerotic plaque in individual vascular territory.**

|  | **Intracranial artery**  (n=3063) | **Extracranial artery**  (n=3047) | **Coronary artery**  (n=3043) | **Subclavian artery**  (n=3009) | **Aortic artery**  (n=3036) | **Renal artery**  (n=3043) | **Ilio-femoral artery**  (n=3048) |
| --- | --- | --- | --- | --- | --- | --- | --- |
| **Overall LE8 scores** |  |  |  |  |  |  |  |
| Low (<60) | Ref. | Ref. | Ref. | Ref. | Ref. | Ref. | Ref. |
| Moderate (60-<80) | 0.44 (0.34-0.55) | 0.81 (0.66-1.00) | 0.62 (0.50-0.76) | 0.66 (0.53-0.82) | 0.42 (0.29-0.61) | 0.54 (0.43-0.68) | 0.44 (0.31-0.63) |
| High (≥80) | 0.27 (0.19-0.38) | 0.61 (0.46-0.79) | 0.38 (0.29-0.49) | 0.32 (0.25-0.43) | 0.20 (0.13-0.29) | 0.22 (0.16-0.31) | 0.18 (0.12-0.26) |
| **Medical LE8 scores** |  |  |  |  |  |  |  |
| Low (<60) | Ref. | Ref. | Ref. | Ref. | Ref. | Ref. | Ref. |
| Moderate (60-<80) | 0.55 (0.45-0.68) | 0.92 (0.77-1.10) | 0.59 (0.49-0.70) | 0.68 (0.57-0.81) | 0.44 (0.34-0.57) | 0.56 (0.47-0.68) | 0.47 (0.37-0.60) |
| High (≥80) | 0.29 (0.21-0.39) | 0.71 (0.58-0.87) | 0.42 (0.34-0.52) | 0.36 (0.29-0.44) | 0.24 (0.18-0.31) | 0.24 (0.19-0.32) | 0.22 (0.17-0.29) |
| **Behavior LE8 scores** |  |  |  |  |  |  |  |
| Low (<60) | Ref. | Ref. | Ref. | Ref. | Ref. | Ref. | Ref. |
| Moderate (60-<80) | 1.07 (0.81-1.42) | 1.10 (0.89-1.37) | 0.86 (0.69-1.07) | 0.77 (0.62-0.97) | 0.84 (0.62-1.14) | 1.01 (0.78-1.29) | 0.71 (0.52-0.97) |
| High (≥80) | 0.82 (0.60-1.11) | 0.83 (0.65-1.05) | 0.88 (0.69-1.12) | 0.67 (0.52-0.85) | 0.67 (0.48-0.92) | 0.88 (0.67-1.16) | 0.65 (0.47-0.89) |

LE8 indicates Life’s Essential 8; OR, odds ratio; CI, confidence interval; Ref., reference.

^a^ The multivariable model was adjusted for age, gender, current drinking, estimated glomerular filtration rate, antiplatelet and anticoagulants drugs.

**Table S3 Associations of LE8 with the presence of atherosclerotic stenosis in individual vascular territory.**

|  | **Intracranial artery**  (n=3063) | **Extracranial artery**  (n=3047) | **Coronary artery**  (n=3043) | **Subclavian artery**  (n=3009) | **Aortic artery**  (n=3009) | **Renal artery**  (n=3043) | **Ilio-femoral artery**  (n=3048) | **ABI ≤0.9**  (n=3045) |
| --- | --- | --- | --- | --- | --- | --- | --- | --- |
| **Overall LE8 scores** |  |  |  |  |  |  |  |  |
| Low (<60) | Ref. | Ref. | Ref. | Ref. | Ref. | Ref. | Ref. | Ref. |
| Moderate (60-<80) | 0.54 (0.36-0.82) | 0.25 (0.12-0.51) | 0.71 (0.56-0.91) | 0.85 (0.62-1.17) | 0.37 (0.22-0.63) | 0.56 (0.38-0.82) | 0.44 (0.35-0.56) | 0.35 (0.18-0.70) |
| High (≥80) | 0.42 (0.23-0.77) | 0.14 (0.03-0.62) | 0.51 (0.36-0.71) | 0.47 (0.30-0.76) | 0.09 (0.02-0.37) | 0.02 (0.003-0.18) | 0.11 (0.07-0.17) | 0.44 (0.19-1.05) |
| **Medical LE8 scores** |  |  |  |  |  |  |  |  |
| Low (<60) | Ref. | Ref. | Ref. | Ref. | Ref. | Ref. | Ref. | Ref. |
| Moderate (60-<80) | 0.73 (0.51-1.05) | 0.42 (0.21-0.87) | 0.73 (0.59-0.90) | 0.65 (0.50-0.84) | 0.47 (0.28-0.81) | 0.58 (0.41-0.83) | 0.49 (0.39-0.61) | 0.95 (0.49-1.83) |
| High (≥80) | 0.30 (0.17-0.55) | 0.06 (0.01-0.44) | 0.56 (0.43-0.74) | 0.33 (0.23-0.49) | 0.21 (0.09-0.51) | 0.19 (0.10-0.38) | 0.27 (0.20-0.37) | 0.72 (0.33-1.60) |
| **Behavior LE8 scores** |  |  |  |  |  |  |  |  |
| Low (<60) | Ref. | Ref. | Ref. | Ref. | Ref. | Ref. | Ref. | Ref. |
| Moderate (60-<80) | 0.97 (0.58-1.63) | 0.31 (0.13-0.75) | 0.83 (0.63-1.08) | 1.04 (0.73-1.49) | 0.41 (0.23-0.74) | 0.82 (0.52-1.28) | 0.63 (0.49-0.81) | 0.28 (0.12-0.62) |
| High (≥80) | 1.08 (0.62-0.88) | 0.40 (0.15-1.05) | 0.73 (0.54-0.99) | 0.95 (0.64-1.41) | 0.28 (0.13-0.60) | 0.54 (0.31-0.91) | 0.41 (0.30-0.55) | 0.26 (0.11-0.62) |

LE8 indicates Life’s Essential 8; OR, odds ratio; CI, confidence interval; ABI, ankle-brachial index; Ref., reference.

^a^ The multivariable model was adjusted for age, gender, current drinking, estimated glomerular filtration rate, antiplatelet and anticoagulants drugs.

**Table S4** **Subgroup analysis of LE8 with the presence and extent of atherosclerotic stenosis in participants without history of ASCVD.**

| **Categories** | **n/N (%)** | **Presence of atherosclerotic stenosis** | |  | **Extent of atherosclerotic stenosis** | |
| --- | --- | --- | --- | --- | --- | --- |
|  |  | **OR (95%CI)** | ***P* value** |  | **cOR (95%CI)** | ***P* value** |
| **Overall LE8 score** |  |  |  |  |  |  |
| Low (<60) | 243/440 (55.2) | Ref. |  |  | Ref. |  |
| Moderate (60-<80) | 746/1975 (37.8) | 0.54 (0.43-0.67) | <0.001 |  | 0.50 (0.41-0.62) | <0.001 |
| High (≥80) | 123/556 (22.1) | 0.30 (0.22-0.39) | <0.001 |  | 0.26 (0.20-0.34) | <0.001 |
| **Medical LE8 score** |  |  |  |  |  |  |
| Low (<60) | 433/861 (50.3) | Ref. |  |  | Ref. |  |
| Moderate (60-<80) | 493/1339 (36.8) | 0.58 (0.48-0.69) | <0.001 |  | 0.55 (0.46-0.65) | <0.001 |
| High (≥80) | 186/771 (24.1) | 0.35 (0.28-0.43) | <0.001 |  | 0.31 (0.25-0.39) | <0.001 |
| **Behavior LE8 score** |  |  |  |  |  |  |
| Low (<60) | 243/511 (47.6) | Ref. |  |  | Ref. |  |
| Moderate (60-<80) | 507/1372 (37.0) | 0.72 (0.57-0.90) | 0.005 |  | 0.71 (0.57-0.88) | 0.002 |
| High (≥80) | 362/1088 (33.3) | 0.65 (0.51-0.84) | <0.001 |  | 0.61 (0.48-0.77) | <0.001 |

ASCVD indicates atherosclerotic cardiovascular disease; LE8, Life’s Essential 8; OR, odds ratio; CI, confidence interval; ABI, ankle-brachial index; Ref., reference.

^a^ The multivariable model was adjusted for age, gender, current drinking, estimated glomerular filtration rate, antiplatelet and anticoagulants drugs.

**Table S5 Receiver operator curve of LE8 for the presence of atherosclerotic plaque and stenosis.**

| **Model** | **AUC** | **95%CI** |
| --- | --- | --- |
| **Presence of atherosclerotic plaque** | | |
| Overall LE8 score | 0.651 | 0.620-0.682 |
| Medical LE8 score | 0.678 | 0.645-0.711 |
| Behavior LE8 score | 0.565 | 0.528-0.602 |
| **Presence of atherosclerotic stenosis** | | |
| Overall LE8 score | 0.600 | 0.583-0.617 |
| Medical LE8 score | 0.612 | 0.593-0.631 |
| Behavior LE8 score | 0.553 | 0.533-0.572 |

LE8 indicates Life’s Essential 8; AUC, area under the curve; CI, confidence interval.

**SUPPLEMENTAL FIGURE LEGENDS**

**Fig. S1 Distribution of LE8 categories with** **the presence of atherosclerotic plaque** **in individual vascular territory.** LE8 indicated Life’s Essential 8.

^a^LE8 scores, medical score and behavior score were classified as low (<60), moderate (60-<80) or high (≥80) group.


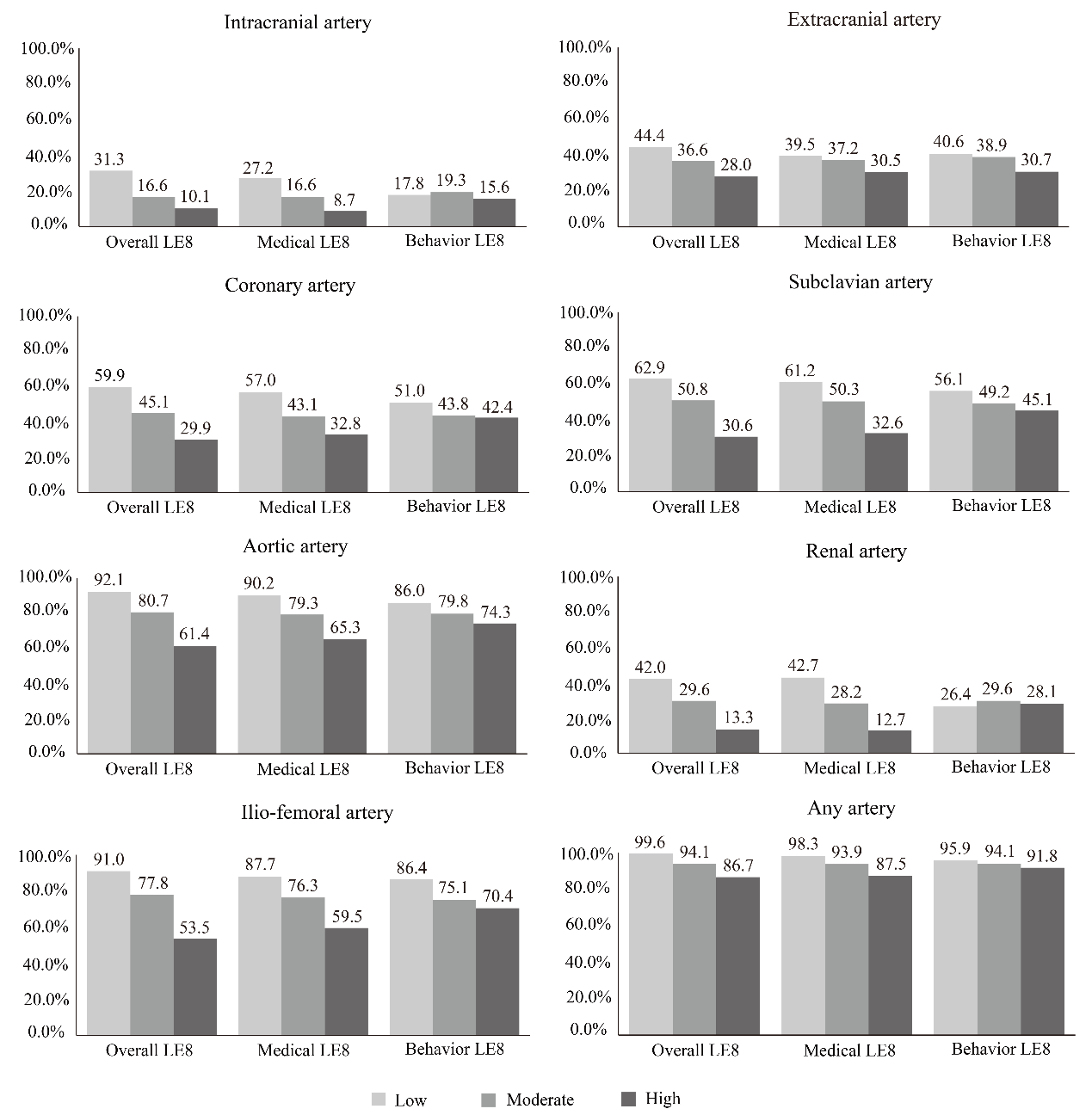


**Fig. S2 Distribution of LE8 categories with the presence of atherosclerotic stenosis in individual vascular territory.** LE8 indicates Life’s Essential 8.

^a^LE8 scores, medical score and behavior score were classified as low (<60), moderate (60-<80) or high (≥80) group.


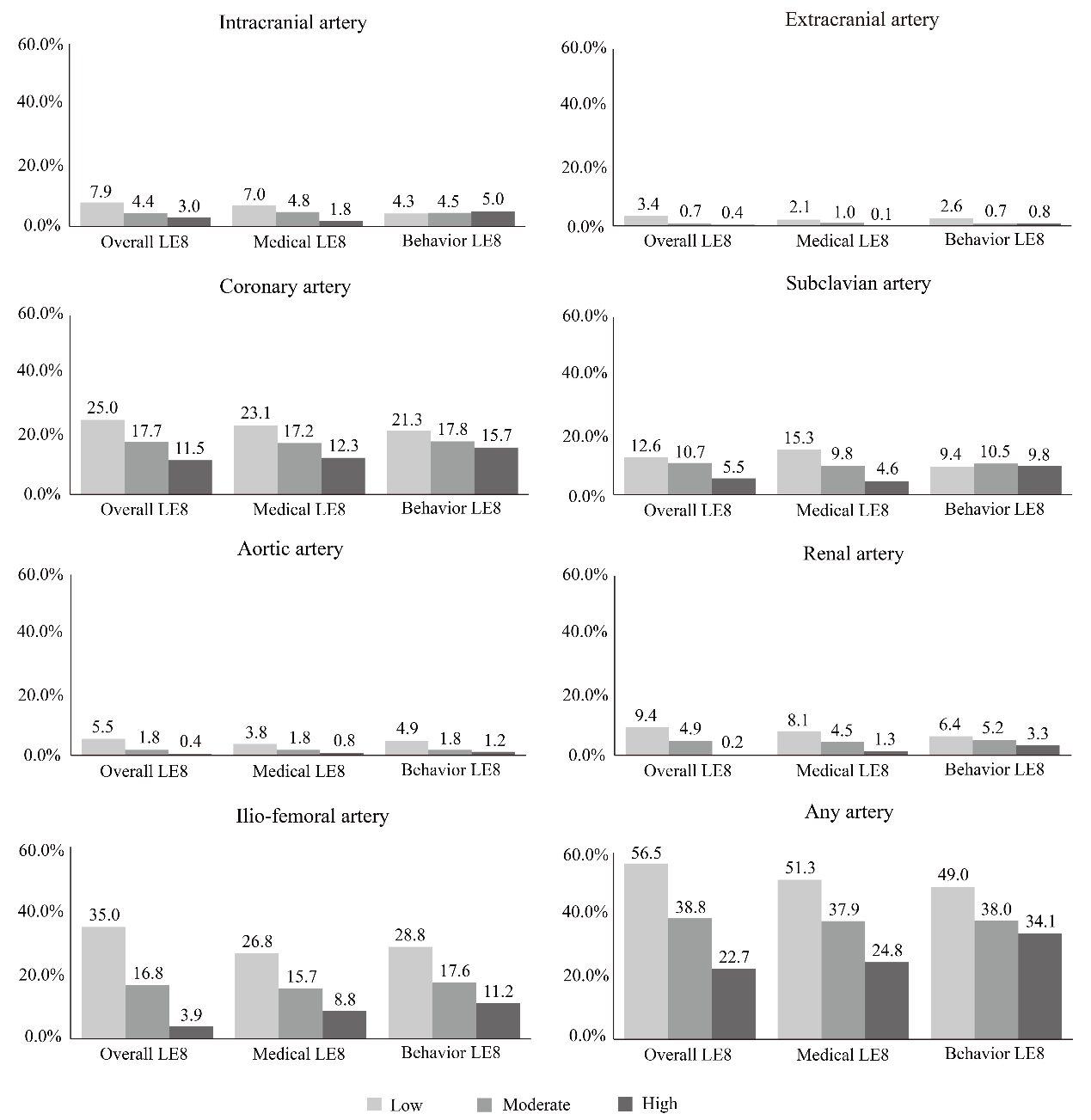


**Fig. S3** **Values of mean scores for LE8 scores with presence of plaque and stenosis.** LE8 indicated Life’s Essential 8.


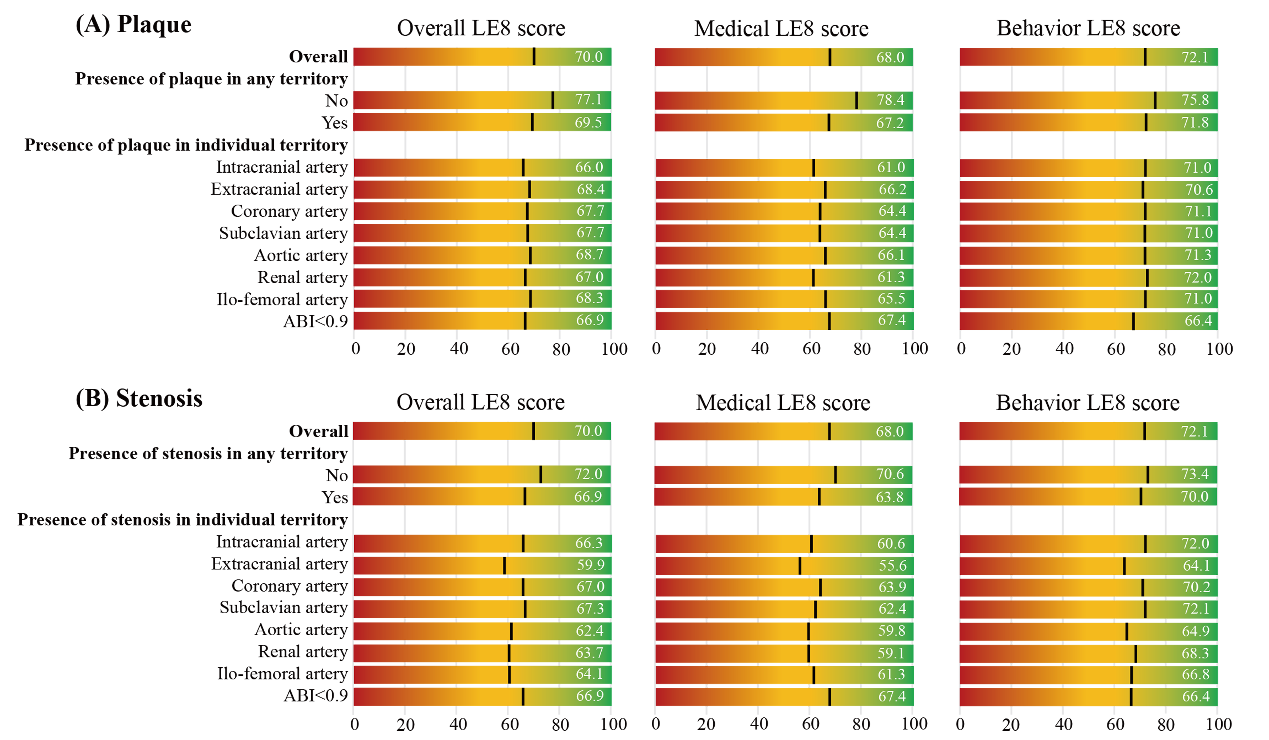

Supplement: Supplementary file 1 — Supplementary Material 1 [file 12877_2024_5119_MOESM1_ESM.docx]
